# Potential Pathways and Pathophysiological Implications of Viral Infection-Driven Activation of Kallikrein–Kinin System (KKS)

**DOI:** 10.3390/v16020245

**Published:** 2024-02-03

**Authors:** Sharton Vinícius Antunes Coelho, Fabiane Messner Augusto, Luciana Barros de Arruda

**Affiliations:** Departamento de Virologia, Instituto de Microbiologia Paulo de Góes, Universidade Federal do Rio de Janeiro, Rio de Janeiro 21941-902, Brazil; messner.fabiane@gmail.com

**Keywords:** dengue, SARS-CoV-2, hantavirus, bradykinin, kininogen, factor XII

## Abstract

Microcirculatory and coagulation disturbances commonly occur as pathological manifestations of systemic viral infections. Research exploring the role of the kallikrein–kinin system (KKS) in flavivirus infections has recently linked microvascular dysfunctions to bradykinin (BK)-induced signaling of B2R, a G protein-coupled receptor (GPCR) constitutively expressed by endothelial cells. The relevance of KKS activation as an innate response to viral infections has gained increasing attention, particularly after the reports regarding thrombogenic events during COVID-19. BK receptor (B2R and B1R) signal transduction results in vascular permeability, edema formation, angiogenesis, and pain. Recent findings unveiling the role of KKS in viral pathogenesis include evidence of increased activation of KKS with elevated levels of BK and its metabolites in both intravascular and tissue milieu, as well as reports demonstrating that virus replication stimulates BKR expression. In this review, we will discuss the mechanisms triggered by virus replication and by virus-induced inflammatory responses that may stimulate KKS. We also explore how KKS activation and BK signaling may impact virus pathogenesis and further discuss the potential therapeutic application of BKR antagonists in the treatment of hemorrhagic and respiratory diseases.

## 1. Introduction

Viral diseases involve complex virus–host interactions, with inflammatory and vascular disturbances frequently associated with clinical syndromes. However, there is limited knowledge about the role of proteolytic cascades in the interplay between virus replication in endothelial and inflammatory cells and infection-associated vascular pathology. Hemorrhagic viral infections are striking examples of diseases in which vascular disturbances may lead to fatal outcomes, marked by excessive vasodilation and increased vascular permeability. These alterations result in blood or plasma extravasation into tissues, tissue edema, and hypotension [1,2,3]. Additionally, infection-driven hyperinflammation affects the microvasculature, leading to systemic syndromes, as recently evidenced in COVID-19 patients [4,5]. 

The contact system serves as the activation starting point for both the intrinsic coagulation pathway and the kallikrein–kinin system (KKS), representing an intimate connection between coagulation and intravascular inflammation [6]. The prothrombogenic and proinflammatory responses resulting from the converging action of these cascades contribute to the pathogenesis of several infectious diseases caused by intracellular protozoa [7,8,9,10], yeasts [11], Gram-positive bacteria [12,13], or viruses [14,15].

Evidence of KKS activation has increasingly been reported during infection by different viruses, especially those associated with hemorrhagic and respiratory diseases, such as dengue (DENV), hantavirus, and SARS-CoV-2 [15,16,17,18]. Virus-induced cell death, as well as activation of innate immune cells, leads to the release of potential KKS activators and are the main mechanisms proposed to stimulate this proteolytic cascade [19,20,21,22]. KKS activation ultimately generates kinin’s vasoactive peptides. Signaling through kinin receptors affects the vascular tone and promotes vascular permeability, contributing to edema and the infiltration of inflammatory cells into tissues [23,24]. Despite the recent use of pharmacological modulators of KKS in experimental models and clinical trials [25,26,27,28,29], there are still many steps that need to be molecularly unveiled to further understand the interplay between virus infection and KKS-mediated vascular disturbances.

Here, we will review the pathways of contact system and KKS activation that are potentially relevant for viral pathogenesis and discuss the available therapeutic interventions, especially those related to hemorrhagic and respiratory viruses.

## 2. The Kallikrein–Kinin Proteolytic System and Bradykinin Effects

Kinins are the main product of KKS activation acting on the microvasculature and specific tissues. The metabolic cascade resulting in BK production is initiated by the cleavage of high- or low-molecular-weight kininogen (HMWK and LMWK) by activated serine protease kallikrein (PKa), present in intra- and extravascular compartments [30,31]. Plasma kallikrein cleavage of HMWK releases the nonapeptide BK (Arg-Pro-Pro-Gly-Phe-Ser-Pro-Phe-Arg), while tissue kallikrein acts on LMWK, generating kallidin or LysBK (Lys-Arg-Pro-Pro-Gly-Phe-Ser-Pro-Phe-Arg) [32]. These kinins can be further degraded by M- or N-type carboxypeptidases, which remove an arginine (Arg) from their C-terminal region, generating des-Arg-BK and Lys-des-Arg-BK [33,34].

Kinins exert their biological functions through the activation of the GPCRs B1R and B2R [35,36]. BKR signaling is associated with vasodilation, hypotension, increased vascular permeability, edema formation, angiogenesis, and pain [23,24], usually present during several viral infections. 

BK and des-Arg-BK or Lys-des-Arg-BK are the preferential agonists of B2R and B1R, respectively. B2R is constitutively expressed in various tissues, including vascular endothelium and smooth muscle, and may also be upregulated under injury or infectious conditions [14,15,37,38]. On the other hand, B1R is barely detected in tissues under physiological conditions but is upregulated in vascular cells after activation of NF-kB or c-Jun and p38 MAP kinases (MAPK) [39]. In this sense, B1R expression is stimulated by IL-1β, TNF, and IFN-γ [40,41,42] and may be fostered in inflammatory and pathological circumstances, such as tissue or vascular injuries, ischemia, and diabetes, as well as upon infection by certain pathogens, including viruses [14,15,43,44,45].

The intracellular signals triggered by B1R and B2R mostly rely on the activation of the Ca^2+^/calmodulin pathway, in association with ERK MAPK and PI3K/PKA stimulation. Those promote increased activity or expression of endothelial and inducible nitric oxide synthases (eNOS and iNOS) and NO production [46,47], as well as production of VEGF and activation of EGFR [48,49]. BKR-induced ERK and NF-kB signaling also result in increased expression of cyclooxygenase2 and production of prostaglandins and prostacyclin [50]. Additionally, B2R signaling stimulates the release of tissue plasminogen activator (tPA) and inhibits platelet aggregation [51,52]. Finally, NF-kB and PI3K/Akt promote upregulation of anti-apoptotic proteins, apoptosis inhibition, and cell proliferation [14,53,54,55,56]. Importantly, activation of B2R, but not of B1R, induces its rapid desensitization and internalization; therefore, B2R usually induces an acute response, whereas B1R may produce a more sustained one [57,58,59]. 

## 3. Interplay between Tissue Lesion and Innate Immune Response with KKS Activation

Activation of KKS is initiated by the contact of plasma FXII with negatively charged surfaces, which induce its autocatalytic cleavage, producing the fragment FXIIa [60]. The enzymatically active FXIIa converts HK-bound PK to the active kallikrein (PKa). PKa fuels the system by cleaving more FXII zymogens, in addition to cleaving HK and releasing BK [60,61]. Individuals with hereditary FXII deficiency and FXII-deficient mice present a striking impairment in thrombus formation induced by different stimuli but do not exhibit abnormal bleeding [62,63]. FXI-, PK-, and HK-deficient mice were also protected from thrombosis, implicating both the intrinsic coagulation pathway and KKS as major pathological mechanisms in thromboembolic development [62,63,64]. 

In vitro or ex vivo experimental models of contact system activation are typically performed using anionic compounds such as dextran sulfate (DXS), kaolin, or silica [64,65,66]. It is challenging to determine the exact substrate inducing FXII activation in vivo, but several naturally negatively charged surfaces have been proposed to stimulate the contact system on the vascular endothelium [19,20,21,22]. These may be induced or amplified by pathogen-driven activation of innate immune cells, as will be further discussed in the following sections.

### 3.1. Exposure of FXII Activators after Tissue Injury

**Figure 1:**
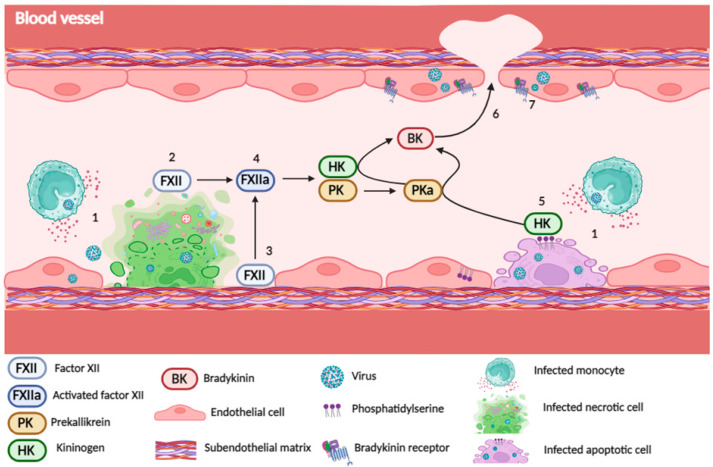
Endothelial cell death and tissue lesion expose potential FXII activators. Inflammatory mediators released by infected circulating cells and by infection of endothelial cells may induce cell death by necroptosis or pyroptosis (1), promoting the release of negatively charged intracellular nucleic acids, which are potential FXII activators (2). Endothelial injury allows the exposure of subendothelial matrix proteins (3), which are also FXII activators. The activation of FXII by these events leads to release of FXIIa (4) and initiation of KKS proteolytic cascade, culminating in the release of BK. In addition, endothelial cell infection may induce apoptosis (5), leading to the exposure of membrane phosphatidylserine, which binds to kininogen, creating a platform that facilitates its cleavage by PKa and fuels BK formation. Increased BK via any of these mechanisms leads to plasma leakage and release of KKS components into the tissues (6). In addition, BKR may be upregulated in infected endothelial cells, amplifying these events (7). Created with BioRender.com (https://help.biorender.com/en/articles/3619405-how-do-i-cite-biorender, accessed on 8 December 2023).

Virus replication in a host cell may induce cell death by different mechanisms, including apoptosis, necroptosis, and pyroptosis. Each of these events results in the expression or release of molecular structures associated with contact pathway activation, as illustrated in Figure 1. HK can directly bind to phosphatidylserine (PS), which is exposed in cells undergoing apoptosis. Incubation of citrated plasma with apoptotic cells resulted in HK cleavage and BK release, but the role of FXII or PK had not been specifically addressed in this model [19]. It has also been proposed that extracellular RNA released by necrotic cells or injured tissues binds to FXII and FXI, contributing to a procoagulative and prothrombotic environment [20]. In addition, the inflammatory response triggered upon infection may affect the microvasculature and promote local or systemic vascular injury. The exposure of the subendothelial matrix allows basement membrane proteins, such as laminin and collagen, to bind and activate FXII, supporting thrombus formation [21,22]. 

### 3.2. Degranulation and Formation of Extracellular Traps (ETs) by Activated Immune Cells as Potential KKS Triggers

About 25 years ago, Kozik and colleagues reported that a mixture of elastase and tryptase, secreted by activated neutrophils and mast cells, could proteolytically cleave kininogen, releasing bradykinin [67]. Elastase secretion by activated neutrophils in vitro and increased serum elastase in patients have been reported during infection of many respiratory viruses, including influenza [68], respiratory syncytial virus (RSV) [69], rhinovirus [70], and SARS-CoV-2 [71]. This increase was correlated with disease severity or exacerbation of asthma-related symptoms, but KKS activation had not been specifically addressed in these models. 

The spike protein from SARS-CoV-2 induced the secretion of tryptase and chymase from human mast cells [72], and enhanced serum levels of these proteases were positively correlated with COVID severity in patients [73]. Furthermore, an increased number of tryptase-expressing mast cells was detected in the alveolar septum of COVID-19 patients, where intra-alveolar edema and increased expression of B1R and B2R were also observed [74]. 

Neutrophil degranulation [75] and increased levels of mast cell-derived proteases [76] were also reported in DENV infection. In addition, DENV induced tryptase secretion by mast cells, which promoted endothelial cell permeability in vitro and in vivo [77]. 

Innate immune cells also secrete extracellular traps (ETs), composed of decondensed chromosomal DNA, histones, and granular proteins, such as defensins, elastase, cathepsins, lactoferrin, and myeloperoxidase (MPO) [78]. ETs were originally described to be released by activated neutrophils (NETs) and function as a mechanism of trapping and eliminating extracellular pathogens [79]. Incubation of human plasma with activated polymorphonuclear cells (PMN) or with PMN-derived DNA resulted in PKa activation, apparently due to the direct interaction of soluble DNA with HK and FXII [80]. Using elegant intravital and in vitro experimental data, von Bruhl and colleagues demonstrated that neutrophils are recruited to venous endothelium and are essential for deep vein thrombus propagations by binding FXII, in a process which depends on the release of DNA/NETs and is amplified by the presence of platelets [81]. On the other hand, Noubouossie et al. (2017) showed that, although purified human DNA or isolated histones activated the contact system, the same effect was not triggered by intact NETs, indicating that further studies are needed to fully understand the physiological role of NETs in thrombus formation in humans [82]. 

**Figure 2:**
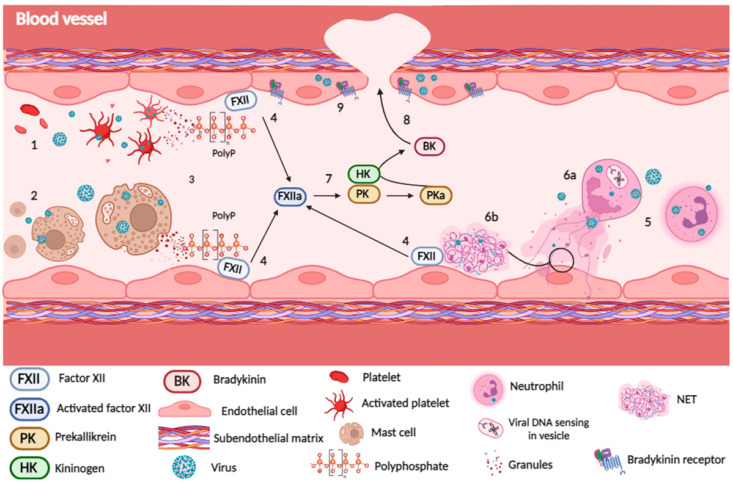
Interplay between innate immune cell stimulation and KKS activation. Virus interaction with platelets (1) and mast cells (2) may trigger cellular activation, leading to increased expression and release of polyphosphates (polyP) (3), which are deposited on endothelial cell surfaces, resulting in the activation and autocleavage of factor XII and release of FXIIa (4). Neutrophil infection and virus sensing (5) also induce the release of granules and NETs (6a, 6b (amplification of the circle)), which contain proteases and negatively charged molecules, such as DNA, RNA, and histones. All these elements are also potential activators of FXII (4). FXIIa activates prekallikrein (PK), generating plasma kallikrein (PKa), which then cleaves its cofactor, kininogen (HK), resulting in the release of bradykinin (BK) (7). BK or des-Arg-BK act through their G-coupled receptors, B1R and B2R, contributing to increased vascular permeability, with cellular and plasma infiltration into tissues (8). Also, infection of endothelial cells may upregulate BKR and contribute to these effects (9). Created with BioRender.com.

Neutrophil activation with NET release can be triggered by different stimuli, including ROS generation upon pathogen recognition receptors’ (PRRs) sensing of pathogen- or damage-associated molecular patterns (PAMPs and DAMPs). In this sense, it was described that HIV and respiratory syncytial virus (RSV) infection stimulate NET release in TLR7/TLR8- and TLR4-dependent pathways, respectively [83,84]. Indeed, soon after NET discovery, several studies demonstrated that they could be released after neutrophil activation by different viruses [85], including HIV [83,86], influenza [87,88], DENV [89], CHIKV [90], RSV [84,91], hantavirus [92], and SARS-CoV-2 [93,94]. On the other hand, the role of ETs released by other cells, such as monocytes, macrophages, and mast cells, during virus infection has been scarcely investigated and is worth further attention. Figure 2 illustrates the potential mechanisms of FXII activation upon neutrophil activation, degranulation, and release of NETs. 

### 3.3. Platelets Derived Surface Polyphosphates as FXII Activators

Müller and colleagues (2009) described that plasma polyphosphates (polyP) secreted by activated platelets promoted FXII activation, leading to PKa-driven HK cleavage and BK formation [95] (Figure 2). They also showed that polyP inoculation resulted in FXII-dependent vascular permeability and coagulation in an in vivo mouse experimental model. Surface polyP present in activated platelets may also activate FXII, favoring thrombus formation [96]. Although the specific role of polyP has never been addressed during virus infections, platelet activation by different virus species has been increasingly demonstrated. Platelets’ exposure to DENV particles or DENV-derived NS1 results in their activation, aggregation, and apoptosis [97,98,99]. Platelet activation was also reported during influenza and SARS-CoV-2 infection, and this activation was related to lung injury and microvascular thrombosis [100,101,102]. Increased polyP expression or release after platelet–virus interactions is worth further investigation and could be related to the contact activation pathway, microthrombosis, and amplification of the inflammatory response.

### 3.4. Virus Receptors/Coreceptors Regulating FXII-Independent KKS Activation

KKS activation may also be regulated by the assembly of HK to the cell surface. It has been proposed that HK binds to endothelial cells, platelets, neutrophils, and monocytes through heparan sulfate and chondroitin sulfate glycosaminoglycans (GAGs) [103,104,105]. HK binding to GAGs protects it from cleavage, whereas degradation of surface GAG restores proteolytic BK generation at cellular surfaces [106]. Since surface GAGs commonly participate in virus adsorption as part of the virus receptor/coreceptor complex or by facilitating virus adhesion [107,108], one can hypothesize that the binding of virus particles or virus proteins could compete with plasma HK for GAG binding or could displace surface HK-GAG, favoring proteolytic BK generation.

## 4. Regulation of KKS Activation and BK Signaling Pathways

Control of KKS activation and BK generation is crucial to prevent hyperinflammatory events with vascular and hemostatic disorders [109]. The serine protease inhibitor (C1INH) blocks the activity of FXIIa, FXI, PKa, and plasmin and is one of the most important inhibitors of KKS [110,111]. Patients with hereditary angioedema (HAE) types I and II present deficient production or function of C1INH and show extensive subcutaneous and submucosal edema affecting the extremities, face, gastrointestinal tract, upper airways, and genital regions [47]. These signs can be controlled through the infusion of C1INH or using inhibitors of BK production or activity, implicating BK overproduction as a major deleterious mechanism in those patients [47,112,113]. FXII activation is also physiologically controlled by α1-antitrypsin and plasminogen activator inhibitor 1 (PAI-1) expressed on endothelial cells [114,115], while PKa can be inhibited by α2-macroglobulin [116]. The pharmacological modulation of these pathways to treat virus-induced diseases is being investigated and will be further discussed. 

BK and its biologically active metabolites can be hydrolyzed by the action of zinc-dependent metalloproteases (Zn^2+^), including angiotensin-converting enzymes—ACE and ACE2—as well as the neutral endopeptidase (NEP) [34,117,118,119]. ACE and NEP convert BK into BK-(1-7) and Lys-BK-(1-7), which are considered inactive peptides [120,121], although active biological functions, independent of B1 and B2 receptors, have been recently suggested [122].

Soluble and cell-associated ACE2 has been associated with the degradation of des-Arg-BK, especially in the lung [118,123]. Using an LPS inhalation challenge murine model, Sodhi and colleagues demonstrated that ACE2-deficient mice presented an exacerbated activation of the des-Arg-BK/B1R axis, culminating in the secretion of proinflammatory chemokines, such as CCL5, MIP2, KC, and TNF, by airway epithelial cells and intense neutrophil infiltration into the lung. These responses were reversed by a B1R antagonist, indicating a new pharmacological strategy to control KKS and des-Arg-BK-mediated pulmonary inflammation [123]. ACE2 is the main receptor of SARS-CoV and SARS-CoV-2 and it has been proposed that virus–ACE2 interaction results in the internalization of the receptor [124]. Therefore, it is possible that SARS-CoV-2 infection results in enhanced availability of des-Arg-BK, further contributing to a proinflammatory milieu. 

Finally, innate pathogen sensing may positively regulate KKS response by enhancing the expression of B1R and B2R. Activation of TLR3 and TLR4 by LPS and poly (I:C) induced B1R and B2R expression through NF-kB and JNK pathways, increasing smooth muscle contraction in a mouse asthma model [125]. Enhanced BKR expression after DENV replication [15] and in patients with upper respiratory virus infections [38] has also been described, indicating that the interplay between virus genome sensing and BKR expression is worthy of further investigation.

## 5. KKS Activation and Bradykinin Signaling during Systemic Virus Infection

### 5.1. Infection by Hantaviruses

Infection by hemorrhagic viruses is often associated with vascular disturbances, vasodilation, plasma leakage, or hemorrhage, which may culminate in hypotension, shock, and multiple organ failure. Endothelial cells have been considered targets for many viruses’ replication, but a significant cytopathic effect is not always detected in vivo, partly due to the intricacy of accessing the tissue [1,126]. However, alterations in coagulation factors and inflammatory mediators have been largely reported [17,127,128,129,130,131] and might contribute to vascular lesions. 

The Hantaviridae virus family encompasses a number of hemorrhagic viruses, with clinical symptoms that include hemorrhagic fever with renal or pulmonary failure by a yet unknown mechanism [132,133]. An in vitro study with a pathogenic hantavirus strain (HTNV) provided the first precedent that KKS activation and BK signaling increases endothelial permeability in a viral infection [17]. In this work, it was observed that infection of endothelial cells (HUVEC) with HTNV, in the presence of FXII, PK, and HK, resulted in enhanced HK cleavage and BK generation. The addition of KKS elements in the infected cultures also resulted in increased permeability, which was inhibited by FXII and PKa inhibitors, as well as by B2R antagonists. These data motivated the development of a clinical trial using B2R antagonists, which resulted in partial recovery of the respiratory symptoms [27], as will be discussed afterward.

### 5.2. Infection by Arboviruses

The nontaxonomic group of arboviruses comprises families of viruses transmitted by mosquitoes or ticks, largely represented by flavivirus and alphavirus families, as well as others not discussed in this review. Flaviviruses include neurotropic and hemorrhagic viruses, causing a range of clinical manifestations, including mosquito-transmitted ones: dengue (DENV), Zika (ZIKV), yellow fever (YFV), West Nile (WNV), and Japanese encephalitis (JEV) viruses [134].

Dengue infection causes vascular alterations associated with vasodilation and plasma leakage, which may ultimately result in hypotension and shock in the most severe forms of the disease [135,136,137]. The first study of KKS in the context of dengue disease was published by Edelman and colleagues in 1975. The authors demonstrate a significant decrease in the levels of FXII and PK zymogens in plasma from children with dengue hemorrhagic fever (DHF) compared to plasma from patients with unrelated fevers. However, these changes were not reflected in BK concentrations, which were also reduced in the dengue patients. Furthermore, no change was observed regarding the plasma levels of kallikrein inhibitor activity [16]. Another study also reported a decreased BK concentration in the plasma of patients with acute dengue when compared with samples from healthy donors [138]. However, these studies did not investigate whether decreased BK was associated with lower production or enhanced degradation of the peptide. Also, since a longitudinal analysis was not performed, one cannot infer whether this might represent a protective feedback response. Increased mRNA expression of naturally occurring plasma protease inhibitors, including C1INH, was detected in circulating cells from patients with mild dengue fever in comparison with patients with hemorrhagic dengue [139], suggesting that this response may contribute to the control of endothelial damage and vascular permeability. Higher levels of C1INH in plasma from dengue patients versus healthy donors have also been detected [140]. Therefore, the literature remains contradictory in relation to the activation of KKS in dengue, and there were no mechanistic studies that appreciably investigated the role of this pathway in the disease.

Our group has recently demonstrated that exogenous BK potentiated DENV replication in endothelial cells through mechanisms related to B2R signaling, NO modulation, and delayed apoptosis [15]. In addition, virus replication stimulated the expression of B1R and B2R in human brain microvascular endothelial cells (HBMECs). In that work, we also explored the KKS activation status in plasma from patients with different clinical forms of dengue. We demonstrated an enhanced consumption of contact pathway factors (FXII and HK), as well as low PKa enzymatic activity when patients’ plasmas were treated with dextran sulfate (DXS), indicating that KKS activation is triggered early upon infection. Remarkably, mouse treatment with a B2R antagonist reduced the viral load in an in vivo model of intracerebral dengue inoculation, further supporting the idea that DENV may co-opt KKS, which in turn fuels virus replication [15].

We also reported similar findings in experimental infection with Sindbis virus (SINV), a prototype model of alphaviruses [14]. In vitro infection of HBMEC with SINV induced increased expression of B1R and B2R, and the addition of BK stimulated virus replication in a B2R-dependent way. Also, mice infected with SINV in the presence of the B2R antagonist HOE-140 showed a lower viral load when compared to untreated animals, demonstrating that B2R activation is beneficial for this alphavirus replication.

Unlike DENV and SINV, treatment of HBMECs infected with the Zika virus (Asian PE243 and African MR766 strains) using exogenous BK was not sufficient to modulate NO production or cell viability and did not affect viral replication [15]. Thus, triggering different pathways reflects different outcomes even regarding viruses from the same genus.

## 6. KKS Activation and Bradykinin Signaling in Respiratory Viral Infections, Including COVID-19

Increased expression of BK receptor airway epithelial cells or enhanced levels of kinins in nasal secretions have been detected in patients and experimental models of respiratory infections caused by several viruses, including rhinovirus (RV) [38,141,142,143], parainfluenza (HPIV) [129], influenza virus (IAV) [142,144], and SARS-CoV-2 [145,146,147]. Using an in vivo model of IAV (H1N1) infection in ferrets, Barnett and colleagues detected increased levels of kinins in the nasal lavage of infected animals, positively correlating with symptomatology of the disease [144]. Increased kinin concentration in the nasal secretion was also detected in volunteers challenged with human rhinovirus compared to individuals who received a placebo [141]. Kinin concentration was positively correlated with respiratory symptoms, suggesting that BK-mediated airway responses contribute to RV-induced cold [141,142]. Higher expression of B1R was also detected in clinical samples from individuals with upper respiratory viral infections (URIs) compared to noninfected and asymptomatic controls [38]. The authors also demonstrated that treatment of human airway epithelial cells with poly I:C induced an increase in B1R and B2R mRNA expression, indicating that BK response may be modulated by dsRNA sensing [38]. 

BK production and activity have also been associated with PIV3 infection. Guinea pigs injected intranasally with this virus develop airway hyperreactivity to inhaled histamine and show an increased influx of inflammatory cells into lung tissue, which was blocked by the B2 receptor antagonist [129]. Thus, taking the set of studies described, it is possible that respiratory viruses could also co-opt KKS during infection, and the induction of bradykinin release may be related to the appearance of certain clinical manifestations in the course of the infection. 

**Figure 3:**
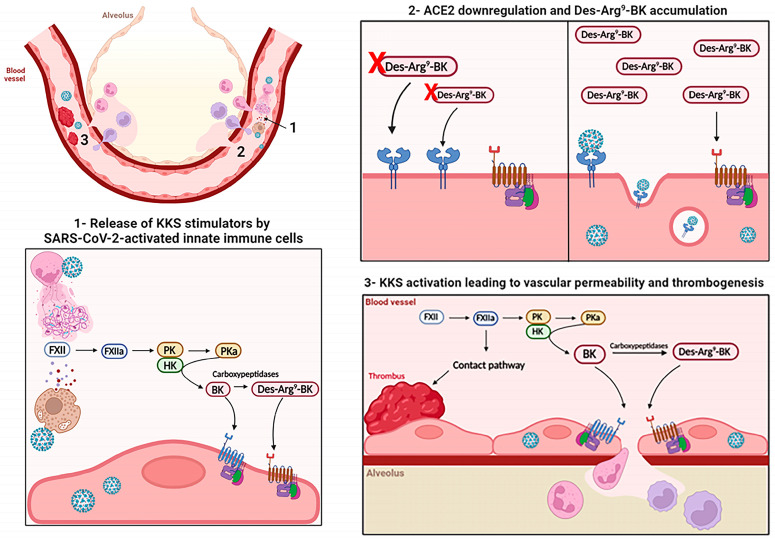
Proposed mechanisms of KKS activation by SARS-CoV-2 leading to increased vascular permeability and thrombogenesis. The upper left panel represents the microcirculation surrounding the alveoli and summarizes the potential mechanisms associated with intravascular activation of KKS, promoting thrombus formation and increased vascular permeability. These mechanisms are detailed in panels 1–3. (**Panel 1.**) Interaction of SARS-CoV-2 with neutrophils and mast cells may induce the release of proteases and polyP; these activate FXII, triggering intrinsic coagulation pathway and KKS.BK and des-Arg-BK signal through B2R and B1R GPCR, respectively (arrows) (**Panel 2.**) ACE2 normally cleaves des-Arg-BK (arrows), controlling its function (left). However, in the context of SARS-CoV-2 infection (right), virus binding to the receptor induces its internalization and reduces its surface expression. ACE2 downregulation leads to increased availability of des-Arg-BK, which signals through B1R, promoting vascular permeability. (**Panel 3.**) Activation of FXII/contact pathway induces thrombus formation and vascular obstruction, contributing to local and systemic vascular syndromes. Also, FXII/HK activation ultimately leads to BK/des-Arg-BK generation, promoting vascular permeability through B2R/B1R signaling (arrows). This figure was created with BioRender.com.

Notably, mechanistic studies regarding KKS activation had not been extensively appreciated until the emergence of COVID-19. Severe COVID-19 is marked by alveolar edema and infiltration of myeloid and PMN cells in the lung tissue [4,5,148,149]. SARS-CoV-2-infected individuals often present coagulative disorders and thromboembolic manifestations [94,102,150,151], and all these events have been associated with increased vascular permeability and inflammatory response. Therefore, thrombus formation through activation of the contact pathway and kinin-mediated increased vascular permeability are potential pathways associated with COVID-19 pathogenesis, as summarized in Figure 3. 

First, the description of ACE2 as a cellular receptor for both SARS-CoV and SARS-CoV-2 highlighted the idea that renin–angiotensin system (RAS) and KKS activation could modulate or be affected by these viruses’ replication [152,153]. Spike proteins from both SARS-CoV and SARS-CoV-2 induced downregulation of surface ACE2 expression in cell lines [124,154]. Accordingly, SARS-CoV-2 replication in permissive cell lines, as well as transfection of those cells with a spike expression vector inhibited ACE2 expression [154,155]. Reduced ACE2 was also detected in the bronchial epithelial cells of a COVID-19 patient, in the lungs of hACE2 transgenic mice infected with SARS-CoV-2, and in the lungs of wild-type infected with SARS-CoV [124,154]. It was then hypothesized that a lower ACE2 availability would result in an enhanced concentration of angiotensin II and des-Arg9-BK, compromising the RAS and KKS in the lungs (Figure 3, panel 2). First, a higher Ang II/Ang1-7 ratio would diminish NO production by the vascular endothelium, favoring vasoconstriction and prothrombogenic events. Second, impaired degradation of des-Arg-BK could enhance local B1R signaling, inducing vascular permeability, fluid extravasation, and immune cell infiltration into the lung [150]. Indeed, increased plasma levels of angiotensin II were detected in COVID-19 patients [124], which is consistent with the hypothesis of lower ACE2 availability, but RAS modulation will not be discussed in this review.

In contrast, differential gene expression analysis in the bronchoalveolar lavage (BALF) cells from COVID-19 patients and infection of lung organoids with SARS-CoV-2 demonstrated an increased expression of ACE2 [145,155]. In the lung organoid model, Xu and colleagues demonstrated that increased ACE2 was associated with enhanced activation of receptor-interacting serine/threonine protein kinase 1 (RIPK1). Pharmacological inhibition of RIPK1 not only reduced ACE2 expression but also the levels of inflammatory mediators. Also, activation of RIPK1 was largely detected in macrophages and neutrophils present in the lung tissues of deceased COVID patients. It is possible, therefore, that ACE2 expression is differentially modulated in SARS-CoV-2-infected cells and the bystander ones. However, this hypothesis requires further investigation.

In the BALF RNA seq analysis, B1R and B2R were also upregulated, whereas ACE expression was reduced [145]. Since reduced ACE favors an enhancement of unmetabolized BK, the study raised the idea of a BK storm inducing increased vascular permeability, hypotension, and plasma leakage in the lung. 

Recent clinical data have supported this hypothesis. Elevated concentrations of tissue kallikrein and BK-derived metabolites were detected in the BALF of COVID-19 patients [146]. Also, increased consumption of plasma FXII, PK, and HK was evidenced in a small COVID-19 patient cohort [18]. In another study, significant increases in plasma levels of intact and cleaved HK, as well as kallikrein:C1 and FXIIa:C1 complexes, were detected in hospitalized patients with COVID-19, suggesting enhanced activation of KKS [156]. KKS activation was also observed in a slightly bigger cohort, comprising 63 patients who developed severe COVID-19 pneumonia [147]. Those patients presented an elevated concentration of plasma HK, with significant enhancement of cleaved HK, when compared to plasmas from healthy subjects. However, enhanced levels of carboxypeptidase N (CPN-1) and BK-(1-8), accompanied by decreased levels of BK, were also detected, indicating BK degradation. Interestingly, increased expression of B1R mRNA in circulating cells was demonstrated, which might favor BK1-8/B1R signaling pathways. It was also observed that the increased BK1-8/BK ratio was associated with inflammatory cytokines (IL-6, TNF, and IL1-β), coagulation markers (D-dimer, fibrinogen, and TF) and lymphopenia [142] and correlated with disease severity. It is important to note, however, that those studies were limited to a few samples, and further investigations with a broader number of patients will be necessary to confirm these findings. 

In an original in vitro study, it was demonstrated that recombinant SARS-CoV-2 structural proteins (S, M, and N proteins) could bind to HK and FXII and induce BK generation when incubated with plasma from healthy donors, suggesting that virus protein expression or deposition in cells may trigger KKS activation [157].

It is conceivable that the activation of inflammatory cells might also contribute to KKS activation due to the secretion of negatively charged metabolites or enzymes with potential HK cleavage activity. In this context, degranulating mast cells, as well as elastase and NET-secreting neutrophils, are potential candidates for KKS amplification during SARS-CoV-2 infection (Figure 3, panel 1). Mast cell degranulation was detected in the lungs of mouse and nonhuman primate models of COVID-19 [158]. Also, increased levels of circulating chymase were detected in hospitalized patients in comparison with healthy donors or individuals with mild disease. Histopathological analyses of lung tissues from fatal cases of COVID-19 indicated a higher frequency of tryptase-positive mast cells, as well as B1R and B2R, in the alveolar septa and perivascular regions when compared to control tissues or tissues from patients who died from H1N1 pneumonia [74,148]. Therefore, mast cell stimulation leading to an increased KKS response is worth investigating in the context of COVID-19 pathogenesis.

Neutrophil infiltration has also been largely reported in the lungs of COVID-19 patients [93,94,149,159,160,161]. Once activated, these cells may secrete elastase and NETs, which are potential stimulators of KKS [81,94]. Englert and colleagues (2021) recently demonstrated an enhanced frequency of activated FXII in the plasmas and lungs of COVID-19 patients and observed that FXIIa colocalized with accumulated NETs in the pulmonary tissue. The authors proposed that increased neutrophil infiltration and activation, in association with impaired NET clearance, might be fostering FXII activation [161]. Another study showed elevated levels of kinin peptides and myeloperoxidase (MPO)–DNA complexes, a NET marker, in the BALF of COVID-19 patients [146]. The concentration of BK-derived metabolites and of MPO–DNA were positively correlated, further corroborating the idea of an interplay between neutrophil secretion and KKS activation during pulmonary COVID-19 disease.

## 7. KKS Involvement and Bradykinin Signaling in Other Viral Infections 

KKS activation and its effects during chronic viral infections, such as those caused by human immunodeficiency virus (HIV), cytomegalovirus (CMV or HHV-5), and hepatitis B virus (HBV), are poorly understood. However, in light of recent findings, especially in the context of COVID-19, an increasing number of studies have been incorporating KKS evaluation, and further data should emerge over the next few years.

Most of those studies addressed the role of BK response in endothelial cells in the stimulation of atherogenesis and atherosclerosis. Individuals seropositive for CMV presented a higher index of coronary artery calcification and showed a decreased response to BK-inducing vasodilation [162]. The authors suggested that CMV infection with reduced BK response might be associated with endothelial dysfunction and increased risk of atherosclerotic burden. Similarly, two independent studies in HIV suggested that tat and nef HIV proteins compromised endothelial functions by decreasing eNOS expression and endothelial responsiveness to BK [163,164]. In this context, impairment of vasodilation via the BK/NO axis by HIV could partially explain the development of heart coronary disease and pulmonary artery disease in HIV-positive patients.

The expression of BKR was also evaluated in a model HBV infection. HBV may cause hepatocellular carcinoma (HCC), and an increased frequency of HCC was reported in Alaska Native individuals, mostly infected with the HBV F1b genotype, which presents specific core mutations. Hayashi and colleagues (2019) used a transgenic mouse model, expressing human hepatocytes, and they observed that the ones infected with genotype F1b presented an increased expression of the BDKRB2 (B2R) gene, associated with greater cell proliferation and HCC development [165]. These data further indicate the many cellular alterations that may be triggered by KKS activation and BK generation upon virus infection.

## 8. Pharmacological Modulation of KKS Activation and BKR

Several drugs have been developed to inhibit KKS-related enzymes or antagonize BKR signaling. Some of them are clinically available or being evaluated in clinical trials. Icatibant (HOE-140 or Fyrazir^®^) is an antagonist of the B2 receptor, which has been shown to prevent BK signaling and control BK-associated inflammatory manifestations [166]. Treatment of C1INH-deficient mice with icatibant was sufficient to reverse increased vascular permeability [167]. In addition, phase II and III studies with this antagonist have been carried out for the treatment of patients who develop HAE type I and II with beneficial clinical results [168,169,170]. It is worth noting that icatibant is currently licensed in over 37 countries, including in Europe and the United States [171].

Regarding virus infection, icatibant administration has been correlated with the recovery of patients infected with Puumala HTNV [25,26,172]. A few preliminary clinical studies have also investigated whether pharmacological modulation of KKS could affect COVID-19 prognosis. In a nonrandomized exploratory study by van Veerdonk and colleagues, ten patients were treated with three doses of 30 mg of icatibant by subcutaneous injection at 6 h intervals and showed improved oxygenation in comparison to nontreated patients [27].

Other pilot trials suggested a clinical benefit when using recombinant C1INH—conestat alfa—in COVID-hospitalized patients [28,173]. Treated patients showed immediate defervescence and oxygen stabilization, as well as a lower viral load. On the other hand, Mansour and colleagues (2020) demonstrated that the treatment of 30 patients with severe COVID-19 using icatibant and conestat alfa did not affect the overall clinical outcome of the patients, although the treatment was safe and associated with increased blood eosinophils and improved lung computed tomography score [174]. Using another approach, phase I studies tested the safety and tolerability of healthy subjects to treatment with a single and escalating dose of a monoclonal antibody against FXIIa, called garadacimab, demonstrating promising initial results [29]. However, future clinical studies will be needed to explore the effects of garadacimab as a treatment strategy for patients with COVID-19.

## 9. Conclusions

KKS is emerging as an important pathway associated with virus-inducing proinflammatory and procoagulant responses. Possibly driven by the well-known effects of kinins during asthma and bronchoconstriction response [175], previous research has demonstrated that virus respiratory infections were associated with increased expression of BKR and high levels of tissue and circulating kinins [38,129,141,142,144]. However, the connection between KKS and virus pathogenesis was not much appreciated until the discovery of ACE2 as a major receptor for SARS-CoV and SARS-CoV-2 [152,153]. Indeed, evidence of KKS activation was detected in the plasma and lungs of COVID-19 patients and correlated with lung inflammation [146,161]. These findings motivated the investigation of KKS modulators as alternative antiviral therapeutics in clinical trials, including B2R antagonist (HOE14/icatibant/Fyrazir), recombinant C1INH (conestat alfa), and anti-FXIIa (garadacimab) [27,28,29,173,174].

Altered levels of circulating intact or activated FXII and HK, as well as bradykinin and C1INH, have also been demonstrated in systemic and hemorrhagic virus infections, such as DENV and HTNV [15,16,138,139,140]. HTNV replication in endothelial cells directly promoted HK cleavage [17]. Also, BKR signaling potentiated alpha and flavivirus replication [14,15]. HTNV-infected individuals have been successfully treated with icatibant in clinical trials [25,26,170], further supporting the therapeutic relevance of controlling KKS activation.

Still, there are few data exploring the therapeutical possibilities of KKS modulation in viral diseases. This may be explained by the biological and technical challenges in experimental investigation, including the limitation of available animal models. The repositioning of drugs licensed for the treatment of hereditary angioedema, which targets excessive BK production, has been hypothesized as an important tool for controlling the symptoms of these infections [167,168]. Therefore, an overall effort of the scientific community for the development of in vitro and in vivo experimental models would be essential to further understand the biological role of KKS in viral diseases and fully explore the therapeutic impact of modulating this pathway. 

## Abbreviations

ACE	Angiotensin-converting enzyme
ACE-2	Angiotensin-converting enzyme 2
B1R/B2R	Bradykinin receptor 1 and 2
BALF	Bronchoalveolar lavage fluid
BK	Bradykinin
BKR	Bradykinin receptor
C1INH	C1 esterase inhibitor
CPN-1	Carboxypeptidase N
DAMP	Damage-associated molecular pattern
DHF	Dengue hemorrhagic fever
DXS	Dextran sulfate
EGFR	Epidermal growth factor receptor
ETs	Extracellular traps
FXI	Factor XI
FXII	Factor XII zimogen
FXIIa	Activated factor II
GAGs	Glycosaminoglycans
GPCR	G protein-coupled receptor
HAE	Hereditary angioedema
HBMEC	Human brain microvascular endothelial cells
HCC	Hepatocellular carcinoma
HK	Kininogen
HMWK	High-molecular-weight kininogen
HTNV	Hantaan virus
HUVEC	Human umbilical vein endothelial cells
KKS	Kallikrein–kinin system
LMWK	Low-molecular-weight kininogen
MAPK	MAP kinases
MPO	Myeloperoxidase
NEP	Neutral endopeptidase neprilysin
NET	Neutrophils extracellular traps
PAI-1	Plasminogen activator inhibitor-1
PAMP	Pathogen-associated molecular pattern
PI3K	Phosphoinositide 3-kinases
PK	Prekallikrein
PKa	Activated kallikrein or plasma kallikrein
PKA	Protein kinase A
PMN	Polymorphonuclear cells
polyP	Polyphosphates
PRR	Pattern Recognition Receptors
PS	Phosphatidylserine
RAAS	Renin–angiotensin–aldosterone system
RAS	Renin–angiotensin system
RIPK1	Receptor-interacting serine/threonine protein kinase 1
RSV	Respiratory syncytial virus
TF	Tissue factor
tPA	Tissue plasminogen activator
